# Chicago Dental Society

**Published:** 1887-05

**Authors:** 


### Chicago Dental Society.

CHICAGO, April 5th, 1887.

*Editor Dental Register :*

Dear Sir : At the annual meeting of the Chicago Dental Society held Tuesday evening, April 5th, the following officers were elected for the ensuing year: President, Dr. J. G. Reid; 1st Vice-President, Dr. J. A. Swasey; 2d Vice-President, Dr. G. H. Bentley; Recording Secretary, Dr. C. N. Johnston; Corresponding Secretary, Dr. W. B. Ames; Treasurer, Dr. E. D. Swain; Librarian, Dr. A. W. Harlan; Board of Directors, Drs. G. H. Cushing, E. Noyes, and J. A. Swasey; Board of Censors, Drs. B. L. Rhein, J. W. Wassall, and L. L. Davis.

W. B. AMES, Cor. Secretary.

